# Serving Size Information and Portion Control Cues on Energy-Dense Nutrient-Poor Packaged Snacks in Australian Supermarkets: Current Practices and Opportunities

**DOI:** 10.3390/foods15020397

**Published:** 2026-01-22

**Authors:** Qingzhou Liu, Carla Azzi, Gabrielle De Leeuw, Rebecca Flemming, Hannah Ross-Smith, Jacqueline Ze-ling Tan, Cheuk Wa Wong, Anna Rangan

**Affiliations:** 1 Discipline of Nutrition and Dietetics, Susan Wakil School of Nursing and Midwifery, Faculty of Medicine and Health, The University of Sydney, Sydney, NSW 2006, Australia; qingzhou.liu@sydney.edu.au (Q.L.);; 2 Charles Perkins Centre, The University of Sydney, Sydney, NSW 2006, Australia

**Keywords:** food environment, packaged snacks, visual cues

## Abstract

Packaged discretionary foods that are energy-dense and nutrient-poor are widely available in the current food environment, potentially contributing to overconsumption and excessive energy intake over time. Factors such as on-pack visual cues (for example, front-of-pack image and food units per serving) and structural features (for example, package transparency) have an important role in nudging consumers towards better portion control. As little is known regarding the presence of these features on packaged discretionary foods in the current retail context, this study aimed to examine the presence of such cues on packaged discretionary foods in Australian supermarkets. Six common packaged snacks were selected: ice-cream, chocolate, lollies, sweet biscuits, savoury biscuits and crisps. Data were collected by in-store visits and using retail websites. A total of 1930 products were included; the majority were share packs (n = 1419, 73.5%), followed by multipacks (n = 385, 19.9%) and single packs (n = 126, 6.5%). Less than half of the share pack products (47%) had front-of-pack images aligned with the manufacturer-suggested serving sizes on the Nutrition Information Panel. Structural features, including transparency, partitioning and resealability, were less common and identified in less than 30% of packaged snacks. Overall, the findings showed that on-pack visual cues and structural features are not commonly used for portion control in packaged discretionary foods in Australian retail settings. Opportunities exist to improve on-pack cues and guides to support better portion size decisions.

## 1. Introduction

Excessive consumption of discretionary foods and beverages, those high in saturated fat, added sugars, and/or salt, has been consistently associated with poor diet quality and an elevated risk of chronic conditions such as obesity, cardiovascular disease, and type 2 diabetes [1,2,3]. These foods contribute approximately 35% of total energy intake in children and 31% in adults in Australia [4]. In contrast, recommended discretionary food intake ranges from 0 to 1800 kJ per day, depending on age, height, and activity level, which should be less than 20% of total energy intake [5]. High consumption of these items can displace nutrient-rich foods from the Five Food Groups, such as vegetables, fruits, cereals, milk products/alternatives, and meats/alternatives, leading to poor diet quality, positive energy balance, and potential weight gain [6].

Portion size (that is, the amount of food consumed per eating occasion) is a significant driver of energy intake and plays a critical role in the overconsumption of discretionary foods [7,8]. Numerous factors influence portion size selection, including social norms, individual preferences, and environmental cues such as package size [9]. It is well recognised that larger servings or packages lead to increased consumption, a phenomenon termed the portion size effect [10,11]. Evidence suggests that downsizing package sizes can effectively reduce energy intake, particularly for palatable energy-dense and nutrient-poor foods [10,12].

Beyond size, package design can also shape consumer behaviour. For example, exaggerated on-pack food images that are larger than the suggested serving sizes stated on the nutrition information panel have been shown to increase the amount served and consumed [13,14]. One study found that cereal boxes with exaggerated serving images led participants to pour 20% more cereal compared with the unexaggerated actual serving images, exceeding the recommended portion size by 45% [15]. The effect of on-pack serving size information on portion size selection and consumption has also been investigated, with studies showing conflicting results depending on factors such as serving size display format, consumers’ food literacy, and the perceived healthiness of the product [16,17,18,19,20]. Structural features of the food packaging, apart from providing a primary role in protecting contents and preserving freshness, can similarly impact consumer behaviours [14,21,22,23]. Transparent packaging can increase product visibility and stimulate purchase intent [14,23], while resealable and partitioned packaging (that is, dividing a large serving size into smaller ones) has been shown to support portion control and could help reduce overconsumption [22,24].

Thus, visual and structural cues such as smaller package sizing, on-pack imagery, transparency, partitioning and resealable packaging can potentially offer consumers guidance on portion sizes [21,25,26]. Beyond the mandated display of serving size information, there are no specific regulations governing the display of visual cues on packaged products in Australia [27]. Little is known about packaging types and the presence of visual cues on packaged discretionary foods. Therefore, this study aimed to (1) investigate the use of visual cues and structural features of packaged discretionary foods in the retail setting and (2) examine the associations between package size, manufacturer-suggested serving size, and the presence of visual cues and structural features. While this approach may contribute to a more nuanced understanding of prevailing serving size practices, additional investigation is required to determine whether such cues are intentional and to assess their potential impact on purchasing and consumption patterns.

## 2. Methods

### 2.1. Selection of Products

A cross-sectional audit was undertaken to collect information on serving sizes, on-pack visual cues, and structural features of commonly consumed packaged discretionary foods in the Australian retail setting. Six product categories of packaged discretionary foods were selected: lollies, chocolate, sweet biscuits, savoury biscuits, crisps, and ice-cream (Table 1). These categories were selected as they represented common EDNP snack foods that are available in a variety of packaging types, such as pouches, plastic bags, flow packs of biscuits, tubs for ice-cream, or boxes for sweet and savoury biscuits. All package sizes were included, from single packs and multipacks to share packs. If a range of products contained a variety of flavours but the nutrition information panel (NIP) was otherwise similar, then the ‘original’ or most common flavour of the product was recorded, to reduce duplication. Products sold with other food items (for example, cheese and cracker snacks) were excluded, as they do not accurately reflect the snack category or the typical consumption manner. No ethics approval was required as all data collected in this study were publicly available in-store or online.

### 2.2. Data Entry, Cleaning and Analysis

Data from chain supermarkets (Woolworths, Coles, Aldi, IGA, and Harris Farm) supplemented by health food stores were collected. These companies account for over 83% of the market value in Australia, providing a strong representation of Australian products [28]. Data collection was performed via the companies’ websites, followed by in-store visits for any missing data.

For each product, the brand, product name, package size and type, and energy and serving size information on the NIP were recorded on a Microsoft Excel spreadsheet.

After the removal of duplicates, products were grouped according to the packaging size type. Single packs were defined as individual bags or packaging containing a single specified serving according to the NIP. Share packs were defined as larger bags, boxes or packaging that contained more than one specified serving according to the NIP, for example, ‘Family Pack’, ‘Party Bag’, ‘Twin Pack’, labelled as ‘for sharing’. Multipacks were defined as those with a larger outer pack containing multiple smaller, individually packaged, or single-serve units (this will be referred to as ‘multipack unit’ throughout the manuscript) intended for individual consumption.

For share packs, additional data on the presence of visual cues and structural features were recorded (Table 2). Food images on the front of the pack and the display of food unit numbers were identified as visual cues; resealability, partitioning and transparency were identified as structural features. Front-of-pack images were evaluated against the serving size indicated on the Nutrition Information Panel (NIP), which served as the reference standard. However, reliance on the NIP as a benchmark has inherent limitations, as these values are determined by manufacturers and do not align with the Australian Dietary Guidelines nor with typical consumption patterns. Consequently, substantial variability in serving sizes can occur within the same food category [29,30]. Despite these constraints, examining the alignment between on-pack imagery and NIP serving sizes offers valuable insights, as discrepancies may contribute to consumer confusion and visual bias.

The alignment of front-of-pack images were assessed using one of the following strategies. Firstly, if food units were present on the NIP, then the number of food units displayed on the front of the pack was compared with the number on the NIP. Alignment was noted if the number of food units on display was the same or less than those on the NIP, for example, two squares of chocolate, five lollies, or one sweet biscuit. If the units displayed were notably higher (that is, by one unit for chocolate, lollies, biscuits, or two units for chips or savoury biscuits) compared with the NIP serving size, these were classified as ‘unaligned’. Secondly, if food units were not present on the NIP, then the displayed food image was converted into grams and compared with the NIP-suggested serving size (in grams). Estimation of serving sizes was enabled by food measure guides from the Australian Bureau of Statistics [31]. If the amount displayed was larger by 25% or more, they were classified as ‘unaligned’. Ongoing discussions within the research team were conducted during data analysis to resolve any discrepancies. All decisions were cross-checked by at least two authors (Accredited Practising Dietitians) and all had training in estimating portion sizes.

Descriptive analysis was conducted using IBM SPSS Statistics version 29.0. The package size and manufacturer-suggested serving sizes for each food category were analysed by packaging size types (multipack unit, single pack, and share pack). The number of suggested servings per package for each product was calculated by dividing the package size by the manufacturer’s recommended serving size listed on the NIP. For share pack products, additional analysis of the presence of visual cues and structural features was conducted by food categories.

## 3. Results

Data were collected for 1930 products across six categories of packaged discretionary foods. Overall, the vast majority of included products were share packs (n = 1419, 73.5%), followed by multipacks (n = 385, 19.9%), and single packs (n = 126, 6.5%).

The descriptive analysis indicated that the median package size varied by the packaging size type (multipack unit, single pack, and share pack) (Table 3). The package sizes for multipack units appeared to be the smallest, followed by single packs, then share packs. For share packs, large variations in the number of suggested servings per package was observed. The median number of suggested servings per share pack varied between five to 20 servings, depending on the food type. The minimum ranged from 1.2 servings for share pack crisps, and the maximum was up to 48 servings for share pack sweet biscuits.

Visual cues and structural features on share pack products that may support better portion control are presented in Table 4. Less than half of the share pack products had front-of-pack food images aligned with the manufacturer-suggested serving sizes on the NIP (n = 673, 47%), especially for sweet biscuits (40%), lollies (22%) and ice-cream (13%). Food unit numbers were rarely displayed (5%).

Structural features, including package transparency, partitioning, and resealability, were noted on 20%, 18% and 27% of share packs, respectively. For resealability, all share pack ice-cream products were packaged in resealable tubs, and 57% share pack chocolates had resealable features, while there was a lower proportion in other food categories (<15%). Transparency was more commonly featured on share pack lollies (51%) and savoury biscuits (34%), but it was below 10% in other categories. Partitioning was predominantly present only in share pack chocolates (67%).

## 4. Discussion

This study examined the presence of visual cues and structural features on packaged discretionary foods in Australian supermarkets. Emerging research suggests that select design elements may support portion control by subtly influencing consumer behaviour and reminding them of appropriate portion sizes [21,23,25,32]. Package size is a strong indicator of portion size, with extensive research demonstrating a robust association between package size and consumption, with larger packages promoting greater intake than smaller packages [13,33,34]. In this study, over one quarter of packages were designed for single consumption, either as an individual serving package or units within multipacks. This trend is likely to reflect consumer preferences for convenience, heightened health awareness, and interest in portion control [35]. However, this audit suggested that most products were sold as share packs containing multiple servings, intended for sharing or consumption across several occasions. These findings align with evidence characterising the current food environment as ‘obesogenic’, where excessive consumption from large packages can drive higher energy intake and increase the risk of overweight, obesity, and related chronic diseases [8,10]. Given this susceptibility to overconsumption, incorporating visual and structural cues into larger packages may be an important strategy for promoting portion control [25,32,36].

The visual cues assessed in this study included front-of-pack images, food unit counts, and manufacturer-recommended serving sizes. Most share packs displayed front-of-pack images; however, fewer than half depicted amounts that were aligned with the manufacturer’s suggested serving size on the NIP. Previous studies indicated that images of smaller serving sizes resulted in lower food intake compared with images of larger servings [21]. Contrastingly, the high frequency of exaggerated images on current packaging design might lead to the opposite effect and normalise larger servings [7,25]. Prolonged exposure to such cues could shift consumer perceptions of ‘normal’ portion sizes upward, rendering smaller servings less acceptable [4,7,8]. This finding is in line with existing marketing research that identified food images as a communication tool to attract consumers’ attention and stimulate impulsive purchasing decisions [14,33,37]. For instance, consumers paid most attention to on-pack visual cues to anticipate the taste of a product during the purchase stage prior to opening the package [37,38]. Marketing research has suggested consumers tend to prefer exaggerated food images depicting a larger number of items (e.g., 15 biscuits versus three), as these were perceived to signal a greater quantity within the package [33]. Considering the absence of clear regulations for on-pack serving sizes and visual cues, the appropriateness of the current manufacturer-suggested serving size information remains unclear. From a public health perspective, food images that represent recommended portion sizes could serve as visual cues to promote health-conscious choices and reduce passive overconsumption [8,39,40]. Future initiatives should aim to develop standardised recommendations for on-pack serving size and provide practical guidelines on appropriate portion sizes for implementation by the food industry.

Displaying food unit counts as indicators of serving size provides a clear and practical guide for consumption at a single eating occasion [39]. However, this study observed that only a minority of products included such information, and the rationale behind manufacturers’ decisions regarding on-pack cues and serving size remains unclear. Although clear serving size guidance is widely regarded as a promising strategy for portion control [9,20,40], evidence of its effectiveness in real-world contexts is limited. Most prior research has relied on controlled laboratory manipulations of serving size information, for example, comparing packages labelled as ‘2 servings’ versus ‘4 servings’ [16,34], rather than assessing implementation in retail environments or alignment with household measures [20]. In Australia, serving sizes and corresponding energy values on the NIP are determined by manufacturers, which may lead to unintended consequences for nutritionally vigilant consumers [41,42]. Similar issues exist internationally, where serving size information on packaged food products is mandatory but lacks standardisation [20]. One explanation may be that consumers prioritise energy content over serving size [40,42,43]. Consequently, manufacturers often present smaller suggested serving sizes to display lower energy values (for example, 200 kJ per 20 g serving rather than 1000 kJ per 100 g), thereby promoting sales and encouraging consumption [40,42,43]. Furthermore, the usability of NIP information among general consumers is limited. A large-scale survey reported that more than two-thirds of participants were classified as ‘non-users’ of NIP information, a trend associated with lower income, education, and nutrition literacy [17]. Qualitative evidence also highlights confusion regarding serving size information, with gram-based measures perceived as difficult to interpret and frequently overlooked [9,39,44]. To further illustrate, in the present study, the suggested serving sizes in share-packs were not always a ‘whole number’, for example, a 60 g package of crisps was noted to contain 2.2 servings, which is impractical and can cause consumer confusion. In addition, for share packs, the number of suggested servings per package ranged greatly, from two to 48 servings for sweet biscuits. This large variation may pose challenges for consumers in interpreting serving size information and making informed purchasing decisions [16,29,30,39].

Structural features such as transparency, partitioning and resealability were identified in 20, 18 and 27% of share packs, respectively, although it is unclear whether these features were intentionally designed for portion control, or primarily for presentation and product protection. Emerging evidence suggests that these structural features may exert a stronger influence on consumption than visual cues by physically restricting access to large portions or facilitating self-regulation through more mindful, deliberate eating [22,32].

Research on package transparency, which provides consumers with an authentic view of the food inside, has yielded mixed findings. One study reported that individuals consumed more from transparent packaging than opaque packaging for small palatable discretionary foods (for example, lollies and confectionery), whereas the opposite effect was observed for larger-unit foods (for example, cookies) as regulation of intake was easier with see-through packaging [45]. This paradoxical effect has been explained by Deng et al., who proposed that transparent packaging exerts two opposing effects on consumption [45]. First, it can enhance food salience, with attractive foods increasing visual hunger [46], leading to higher consumption (salience effect). Second, it can facilitate monitoring of food intake, reducing consumption (monitoring effect) [45]. The net effect appears moderated by food characteristics such as unit size and visual appearance [45]. For small, visually attractive foods, the monitoring effect is low, allowing the salience effect to dominate, leading to higher intakes from transparent packaging than from opaque packaging [45]. Conversely, for larger foods, the monitoring effect predominates, reducing consumption. To date, no further studies have confirmed this paradoxical effect in discretionary foods.

Partitioning, the presence of a physical barrier separating servings, has been examined in limited studies. In one experiment, participants consumed more chocolates when presented in aggregated form compared to individually wrapped (partitioned) chocolates [47]. Eighty-two percent of participants in the aggregate condition consumed all chocolates within two days, compared to 45% from the partitioned condition [47]. Similarly, Van Dellen et al. found that separated single-unit food items were consumed at a lower rate than unpartitioned whole food items [48]. For example, discontinuation of intake after the fourth piece occurred more frequently with partitioned chocolate caramels (21%) than with unpartitioned caramels (7%) [48]. Partitioning is hypothesised to introduce a small transaction cost, requiring consumers to tear, break, or open a barrier, creating a decision point for consumers [47]. At this “decision point”, consumers who are trying to control consumption can evaluate whether to continue and therefore shift consumption from automatic mode to deliberative mode. This mechanism can enhance self-control and promote mindful eating, reducing the risk of overconsumption of snack foods. Additional benefits of partitioning include maintaining hygiene and convenience, although concerns have been raised surrounding increased plastic waste and over-packaging [39].

Evidence on resealable packages and portion control is also emerging. In one study, participants consumed less from resealable packages of palatable, energy-dense snacks compared to non-resealable packages while watching a movie [22]. This effect persisted over six days, with resealable packages limiting the amount consumed per eating occasion, although the frequency of occasions remained unchanged [22]. It is hypothesised that resealable packaging promotes self-regulation by imposing restrictive consumption norms, leading consumers to perceive it as inappropriate to consume the entire package in one sitting [22,49]. Saving part of the product for later may reduce perceived appropriate portion size and encourage more deliberate consumption [47]. Both resealability and partitioning were rated as important factors in determining portion size, although rated lower than package size and energy content in semi-structured interviews among young adults [32]. Qualitative studies in the UK and Australia report high consumer acceptability of resealable packaging, citing convenience, environmental benefits, and assistance in moderating intake [32,39].

Moving forward, helping consumers make more informed and appropriate portion size selections is a key step toward reducing discretionary food intake [11,50,51]. Qualitative evidence has shown consumers would value more guidance in choosing appropriate portion sizes, for example, by having clear portion size recommendations for discretionary foods, together with consistent public health messaging [9,39]. The incorporation of visual cues such as quantifiable portion size guidance images on packages, as well as structural features such as resealability of share packs, can also be helpful, although more research on their impact on actual consumption in day-to-day settings is needed [22,25]. Additionally, more rigorous environmental-level interventions such as reducing the default servings may be necessary to improve the food environment [9,39,52]. Consumer receptiveness to such measures has been noted, while practical challenges should be carefully considered to ensure feasibility and sustainability [9,39,52]. For instance, a proportional pricing strategy, rather than the current value-sizing strategy, was considered necessary to counter value-for-money perceptions and encourage the purchase of smaller serving sizes [39]. Other potential barriers, including increased manufacturing costs and the potential for additional plastic waste associated with smaller packages, must also be acknowledged and addressed [51].

Future collaborative efforts among health professionals, policymakers, retailers, and food manufacturers are thus essential to develop and implement practical, effective solutions that balance public health objectives with economic and environmental considerations [12,51,53]. One notable example is the Healthy Food Partnership, a collaboration between the government, food industry and public health experts. This initiative has introduced a voluntary guide to assist manufacturers in reducing serving sizes of discretionary foods sold at food outlets and retail [54]. While voluntary approaches can foster industry engagement, they require robust monitoring and evaluation frameworks to assess uptake and impact, and to identify areas for improvement. Existing industry-led initiatives, such as the Be Treatwise campaign used in the confectionery sector, can also be helpful [55,56]. This initiative uses clear on-pack labelling and has standardised serving size information on the NIP to 25 g, aligning with the Australian Dietary Guidelines’ standard serve of 600 kJ for discretionary foods [55,56]. A high participation rate (93%) among confectionery manufacturers indicates strong industry engagement [57], yet the absence of formal evaluation studies limits understanding of its effectiveness.

### Strengths and Limitations

The findings of this study provide insight into the use of visual cues and structural features in the current Australian retail contexts that support portion control. Considering the cross-sectional nature of the collected data, limitations should be acknowledged. The included data only represent six categories of common packaged discretionary foods. There may be missing data as seasonal products were excluded, and product availability depended on the days and locations of visits. However, a comprehensive online and in-store search was conducted, and all data were cross-checked to ensure accuracy and to minimise bias. It should be acknowledged that the current NIP in Australia is determined by food manufacturers rather than by government regulatory bodies. There is a lack of standardisation in suggested serving size information, and large variations within the same food category have been noted [30,43]. Thus, the methodological approach to determine the alignment between front-of-pack food images and suggested serving sizes may be prone to misinterpretation. Nevertheless, all results were cross-checked, and agreement was reached between at least two dietitians trained in visualising portion sizes to determine the final findings.

## 5. Conclusions

Visual cues and structural elements that support portion control are largely absent in current Australian retail environments. Introducing clearer visual indicators alongside standardised portion size recommendations could offer practical tools to help consumers make more appropriate portion choices. While emerging evidence suggests these strategies can positively influence purchasing and consumption behaviours, further research is needed to confirm their effectiveness in real-world settings. Collaborative engagement across sectors, involving health professionals, public health bodies, retailers, and food manufacturers, will be required to improve on-pack labelling and visual cue practices. Implementing such measures has the potential to enhance informed decision making, reinforce moderation messages, and contribute to creating a food environment that aligns with broader public health objectives.

## Figures and Tables

**Table 1:** Inclusion criteria of packaged discretionary foods.

Product Category	Description
**Ice-cream**	Ice-cream products, including tubs, sticks, cones, bites.
**Chocolate**	Solid and filled chocolate products, including bars, blocks, bites; white, milk or dark chocolate, excluding chocolate-coated products (for example, chocolate-coated fruit).
**Lollies**	Hard candies, gummies and jellies, excluding lolly products made with artificial sweeteners.
**Sweet biscuits**	All ready-to-eat sweet biscuits, including products that are coated or uncoated, filled or unfilled.
**Savoury biscuits and crackers**	All ready-to-eat savoury biscuits or crackers, including products with savoury flavours or savoury fillings. Crackers and packaged breadsticks are included.
**Crisps**	Products typically made from fried, baked, or air-fried vegetables such as potatoes, corn, and pulses such as chickpeas. Pretzels and popcorn are included as their packaging and consumption manner commonly resemble that of crisps.

**Table 2:** Characteristics of visual cues and structural features for portion control of packaged discretionary foods.

	Description
**Visual cues**	
Front-of-pack food image	The presence of a food image on front of pack. These images were compared with the suggested serving size on the NIP ^a^ to check alignment ^b^.
Food unit numbers displayed	Displaying the number of food units on front of pack as the suggested serving size. For example, suggested serving size = 2 lollies
**Structural features**	
Package size	Categorised as single pack, share pack, or multipack unit (definitions in Methods).
Transparency	Packaging that is clear or ‘see-through’ that allows for the contents inside to be seen without being opened, either fully or partially.

^a^ NIP: Nutrition Information Panel. ^b^ Alignment: This was assessed by comparing the number of food units or estimated grams between images and NIP serving size. Details of the assessment process are presented in Section 2.2.

**Table 3:** Package sizes, manufacturer-suggested serving sizes, and number of suggested serving sizes per package, by food category.

	Type of Packaging ^a^	Number of Products	Package Size (g)	Manufacturer Suggested Serving Size (g)	Number of Suggested Serving Sizes per Package
			**Median (Min–Max)**	**Median (Min–Max)**	**Median (Min–Max)**
**Ice cream ^b^**	Multipack unit	141	72 (33–102)	72 (33–102)	1.0 (1.0–1.0)
	Share pack	134	636 (275–1880)	69 (42–140)	10.0 (2.5–40.0)
**Chocolate**	Multipack unit	81	18 (6–38)	18 (6–38)	1.0 (1.0–1.0)
	Single pack	54	45 (12–60)	45 (12–60)	1.0 (1.0–1.0)
	Share pack	287	164 (32–620)	25 (8–40)	6.6 (1.6–24.8)
**Lollies**	Multipack unit	23	12 (3–26)	12 (3–26)	1.0 (1.0–1.0)
	Single pack	8	29 (12–50)	29 (12–50)	1.0 (1.0–1.0)
	Share pack	252	190 (20–1000)	25 (0.5–170)	8.8 (1.8–50.0)
**Sweet biscuits**	Multipack unit	78	25 (13–44)	25 (13–44)	1.0 (1.0–1.0)
	Single pack	37	47 (21–90)	47 (21–90)	1.0 (1.0–1.0)
	Share pack	349	190 (50–620)	20 (6–57)	9.8 (2.0–48.0)
**Savoury biscuits**	Multipack unit	12	20 (13–25)	20 (13–25)	1.0 (1.0–1.0)
	Single pack	1	24 (N/A)	24 (N/A)	1.0 (N/A)
	Share pack	211	120 (50–420)	21 (5–75)	6.4 (2.0–22.7)
**Crisps**	Multipack unit	50	19 (2–28)	19 (2–28)	1.0 (1.0–1.0)
	Single pack	26	38 (4–75)	38 (4–75)	1.0 (1.0–1.0)
	Share pack	186	125 (20–500)	25 (10–55)	5.0 (1.2–20.0)

^a^ Single packs were defined as individual bags or packaging containing a single specified serving according to the NIP. Share packs were defined as larger bags, boxes or packaging that contained more than one specified serving according to the NIP. Multipack units were defined as those smaller, individually packaged units within a larger outer pack that are intended for individual consumption. ^b^ No single-pack ice-cream data were captured.

**Table 4:** Presence of visual cues and structural features on share packs (n = 1410), by food category.

Food Category	Number of Share Packs	Visual Cues, n (%)		Structural Features, n (%)		
		Food Image Alignment ^a^, n (%)	Front-of-Pack Food Unit, n (%)	Transparency, n (%)	Partitioning, n (%)	Resealability, n (%)
Ice-cream	134	52 (38)	16 (13)	0 (0)	7 (5)	0 (0)
Chocolate	287	261 (91)	175 (61)	47 (16)	14 (5)	192 (67)
Lollies	252	225 (89)	56 (22)	17 (7)	129 (51)	19 (7)
Sweet biscuits	349	310 (89)	140 (40)	9 (3)	35 (10)	11 (3)
Savory biscuits	211	153 (73)	144 (68)	4 (<1)	72 (34)	38 (18)
Crisps	186	161 (87)	142 (76)	0 (0)	19 (10)	0 (0)
Total (mean %)	1419	1162 (82)	673 (47)	77 (5)	276 (20)	260 (18)

^a^ Food image alignment was assessed by comparing the number of food units or estimated grams between images and NIP serving size.

## Data Availability

The original contributions presented in this study are included in the article. Further inquiries can be directed to the corresponding author.
